# Assessment of Physical Activity During Pandemic Periods: Development of the Questionnaire, Determination of Primary Validity, and Psychometric Properties

**DOI:** 10.7759/cureus.56612

**Published:** 2024-03-21

**Authors:** Atahan Turhan, Öznur Büyükturan, Naime Meric Konar, Buket Büyükturan, Ezgi Metin Basat

**Affiliations:** 1 Department of Physical Therapy and Rehabilitation, Kırşehir Ahi Evran University, Kırşehir, TUR; 2 Department of Biostatistics and Medical Informatics, Bandirma Onyedi Eylul University, Bandirma, TUR; 3 Department of Turkish Language and Literature, Kırşehir Ahi Evran University, Kırşehir, TUR

**Keywords:** covid-19, primary validity, scale development, psychometrics, physical activity, pandemic

## Abstract

This study aimed to develop the "Pandemic Period Physical Activity Scale (PPPAS)" to determine the physical activity level of healthy individuals during the pandemic period. Research data were collected from the “Socio-Demographic Questionnaire Form,” “International Physical Activity Short Form,” “Tampa Scale for Kinesiophobia,” “Coronavirus Anxiety Scale,” “Epidemic Anxiety Scale,” “Expert Evaluation Form,” and “Pandemic Period Physical Activity Scale,” exploratory factor analysis revealed that the scale consisted of 3 sub-dimensions and 31 items. Confirmatory factor analysis suggested that the fit indices χ^2^/Df: 2.343; root mean square error of approximation: 0.048; incremental fit index: 0.955; comparative fit index: 0.954; goodness of fit index: 0.912; normed fit index: 0.923; non-normed fit index: 0.950; adjusted goodness of fit index: 0.896; root mean square residuals: 0.060; standardized root mean square residual: 0.047. The total Cronbach Alpha coefficient of the scale was found to be 0.912 while the intraclass correlation coefficient of the scale was calculated as 0.958 (p<0.001). As a result of the analyses conducted, it was concluded that the PPPAS is a valid and reliable measurement.

## Introduction

Epidemic diseases, which are one of the biggest problems in human history, have been seen and fought both globally and regionally. Pandemic is the name given to epidemic diseases that spread globally and over a very wide geographical area, affecting large numbers of people, causing high rates of mortality and morbidity and leading to socioeconomic deterioration in countries [1]. To be declared a pandemic in the current epidemic situation, the basic features such as the spread of the disease in a wide geography, rapid transmission from person to person, high destructive power, high mortality, not being encountered before, and low community immunity to the disease can be counted [2].

The World Health Organization (WHO) office in China was informed about the cases of pneumonia with unknown cause in Wuhan city on December 31, 2019. On January 3, 2020, 44 cases were detected in Wuhan and reported to WHO by the national authorities in China [3]. The patients applied to the centers with certain complaints such as high fever, shortness of breath, and dry cough. When the chest X-ray findings of the patients were examined, bilateral ground glass opacities were observed. In addition, it was stated that the transmission story of the cases was related to a seafood wholesale market in Wuhan, where live animals such as bats, snakes, and badgers were traded [4]. The cause of the disease cases could not be determined and the measures to prevent its spread were insufficient. Because of this, the epidemic spread rapidly and the city of Wuhan became the center of the pandemic. The WHO has announced that it has declared an "International Public Health Emergency of International Concern" on January 30, 2020, due to the increasing spread of the epidemic. Due to the global increase in cases and the unstoppable increase in the rate of spread, it was named coronavirus disease (COVID-19) by WHO on March 11, 2020, and declared as a pandemic [5].

Due to the high mortality and morbidity observed with the rapid spread of COVID-19, restrictions and quarantines were imposed around the world as the pandemic progressed. These restrictions and quarantine practices included increasing the length of stay at home, closing sports facilities and parks, and banning social events and sports activities [6]. These practices caused physical inactivity along with an increase in the amount of time people stayed at home and inactivity. The state of physical inactivity also brought with it many chronic diseases [7,8].

Existing physical activity scales are inadequate for assessing physical activity levels during pandemics. Most existing scales were developed based on normal daily routines and activities and may not accurately assess challenges and changes in physical activity behavior during pandemics. For example, measures based on activities such as commuting to work, going to the gym, or outdoor recreation may not be appropriate if people are confined to their homes or have limited mobility [9,10].

It is necessary to determine the level of physical activity to improve physical activity and prevent diseases related to inactivity during the pandemic period. It is obvious that such a determination necessitates the development of psychometrically qualified measurement tools. For this purpose, it was aimed to develop, validity and reliability of the "Pandemic Period Physical Activity Scale (PPPAS)" to measure the physical activity level of healthy individuals during the pandemic period.

## Materials and methods

Participants

The study consisted of 583 people between the ages of 18 and 65 who volunteered for the study, were able to read and write, had no chronic illnesses (such as neurological and systemic diseases), and had not been diagnosed with COVID-19 or new mutated variants of this virus. According to Kline, the sample size should be at least 10 times the number of articles. In our study, it was decided to apply each item of the scale to at least 10 people. Accordingly, it was calculated that our scale, consisting of 34 questions, should be applied to at least 340 people. The sample size of our scale was 583 people, which was large and consistent with the literature [11].

Study design

This scale development study was carried out at Kırşehir Ahi Evran University School of Physical Therapy and Rehabilitation, Kırşehir, Turkey, between July 10, 2021, and June 10, 2022, with individuals who met the inclusion criteria. Prior to the start of the study, approval was obtained from Kırşehir Ahi Evran University Faculty of Medicine Clinical Research Ethics Committee on June 7, 2021, with decision number 2021-12/139. The importance and purpose of the study were explained by interviewing the participants who will participate in the study and written consent was obtained from the participants with an informed consent form. The study was carried out in accordance with the principles of the Declaration of Helsinki.

Measurements

The Socio-Demographic Questionnaire, International Physical Activity Questionnaire Short Form (IPAQ-SF), Tampa Scale of Kinesiophobia (TSK), Coronavirus Anxiety Scale (CAS), Epidemic Anxiety Scale (EAS) and Pandemic Period Physical Activity Scale (PPPAS) we developed were applied face-to-face to the participants.

Socio-Demographic Questionnaire

In this socio-demographic questionnaire form, participants' name-surname, age, weight, height, gender, marital status, education level, and occupation information were questioned and recorded in the questionnaire form.

International Physical Activity Questionnaire Short Form

The IPAQ-SF was used to determine the physical activity level of the participants. IPAQ-SF consists of four parts and seven questions. It provides information about vigorous activities performed in a minimum of 10 minutes in the last seven days, moderately vigorous activities, and time spent walking and sitting. Metabolic equivalents of task values are taken as a reference when calculating physical activity levels. Physical activity levels are classified into three categories “active,” “minimally active,” and “inactive” [12].

Tampa Scale for Kinesiophobia

The TSK was used to evaluate the participants' fear-avoidance behavior and re-injury anxiety due to pain. The scale consists of 17 items and a 4-point Likert scale (1=strongly disagree, 2=disagree, 3=agree, 4=strongly agree) is used. Questions 4, 8, 12, and 16 are reverse scored and the total score ranges from 17 to 68. The fact that the person's score is high indicates that kinesiophobia is also high [13].

Coronavirus Anxiety Scale

The CAS was used to determine non-functional anxiety levels in individuals during the COVID-19 pandemic period. This scale is a 5-point Likert scale consisting of five items and one factor. Items in the scale are scored as “0=never,” “1=rarely, less than one or two days,” “2=a few days,” “3=more than seven days,” “4=almost every day in the last two weeks” and each item receives a score between 0 and 4 points. The total point value obtained in the scale varies between 0 and 20. A score of 9 and above indicates a high level of anxiety [14].

Epidemic Anxiety Scale

The EAS was used to measure the anxiety caused by epidemics in people. The scale consists of 18 items and 4 factors. In addition, it is a 5-point Likert scale that is graded in the range of “totally suitable for me,” “very suitable for me,” “moderately suitable for me,” “slightly suitable for me,” and “not at all suitable for me.” The lowest total score that can be obtained on the scale is 18, and the highest score is 90. An increase in the total score indicates an increase in epidemic disease anxiety. The total score obtained by individuals from the EAS is “no anxiety” in the range of 18-32, “low anxiety” in the range of 33-46, “moderate anxiety” in the range of 47-61, “high anxiety” in the range of 62-75 and “very high anxiety" in the range of 76-90 [15].

Process of developing a pandemic period physical activity scale

The methods to be followed in the scale development process, which were stated by Cohen et al. in 1996 and by Hambleton et al. in 2004, were taken as references in the creation of the PPPAS [16,17]. The development method of the scale consisted of five main headings.

Determining the Purpose of the Study and Problems

In this study, a literature review was made using the keywords (pandemic, physical activity) at first and the problems were identified. Based on the identified problem, the aim of the study and sub-problems were formed.

Creating the Item Pool (Creating a Draft Form)

Based on the defined and relevant sub-problem, with the keywords "physical activity" and "pandemic," https://scholar.google.com/, https://pedro.org.au/, https://pubmed.ncbi.nlm.nih.gov/ studies were reviewed and the data collection tools used in these studies were examined. The theoretical infrastructure for the measurement tool to be developed with the source research was created. Then, 10 adult individuals suitable for the sample group were asked to write an essay about physical activity during the pandemic process. The statements that attracted attention as a result of the content analysis made on the compositions and the literature review formed the basis of the related Scale Draft Form (SDF). Initially, a 325-item question pool was created in the SDF. There were closed-ended (structured) and open-ended (unstructured) questions to determine the level of physical activity in the SDF. Closed-ended questions were evaluated with a 5-point Likert scale. The answers to the closed-ended questions in the SDF were arranged as “strongly disagree,” “disagree,” “undecided,” agree,” and “strongly agree.”

Getting an Expert Opinion (Creating a Preliminary Application Form)

An expert team of 11 people consisting of physiotherapists and faculty members who have worked in the field of physical activity was established. An Expert Evaluation Form (EEF) was created for this team to evaluate and interpret the items in the SDF in terms of content validity. The Davis technique was used for scoring and evaluation of expert opinions. Experts evaluated the SDF consisting of 325 items. Then the content validity index (CVI) of the items was calculated according to the EEF. At this stage, the number of items was reduced to 65. Afterward, opinions were received from the Turkish Language and Literature Department of the Faculty of Arts and Sciences about the intelligibility of the items in the form and their compliance with Turkish grammar rules. Opinions were received from the Faculty of Medicine, Department of Biostatistics and Medical Informatics regarding the statistical analysis of SDF. In the last stage, the Pre-Application Form (PAF) consisting of 58 questions was created.

Preliminary and Finalizing the Scale

The 58-question PAF, which was developed by taking into account the expert opinions and recommendations, was applied face-to-face to the sample group (221 people) by the researcher. Item analysis was used to evaluate the items of the form with the data obtained as a result of the pre-application, and internal consistency analysis, which evaluated Cronbach's alpha coefficient, was used to determine the consistency. As a result of the analysis of the data obtained with the pre-application of the form, the reliability rate was found to be low. As a result, 21 items were removed from the form and reliability was found to be high as a result of the re-analysis. Later, the questions that created confusion were clarified by taking the opinion of the Turkish Language and Literature Department of the Faculty of Arts and Sciences. Three questions were removed from the PAF by the expert team involved in the development of the scale, as they created confusion. At the end of these processes, the final PPPAS consisting of 34 (30 closed-ended questions and 4 open-ended questions) was created.

Final Scale Implementation

With the creation of the final version of the scale, it was applied to 583 people who were the main sample group target. Equivalent tests of PPPAS, IPAQ-SF 583; TSK, CAS, and EAS were administered to 479 participants. PPPAS was administered to the same 208 participants at 2-3 week intervals.

Statistical analysis

The data obtained in our study were analyzed using IBM SPSS Statistics for Windows, Version 22 (Released 2013; IBM Corp., Armonk, New York, United States) and Lisrel 9.1 programs. The significance level was taken as p<0.001.

Mean standard deviation, median, minimum, and maximum for numerical variables as descriptive statistics; frequency (n) and percentage (%) values were given for categorical variables. Normal distribution was evaluated with the Kolmogorov-Smirnov test. Experts were consulted to evaluate the content validity of the questions prepared by following the scale development stages. Content Validity Indices were calculated using the Davis Technique. In addition, item analysis was applied to the questions in the question pool, and the incomprehensible and inconsistent questions were removed from the item pool. The test-retest method was used as a reliability analysis. The scale questions were re-administered to the participants with an interval of 2-3 weeks. The reliability of the scale was analyzed by evaluating the intraclass correlation coefficients as a result of the first test and the retest. Pearson correlation analysis was used in the parallel forms method to determine similarity with equivalent forms. Explanatory factor analysis (EFA) and confirmatory factor analysis (CFA) were applied to evaluate the construct validity of the scale. Principal component analysis was utilized as a factor extraction method; whilst the varimax Method was used as a factor rotation method. Kaiser-Meyer Olkin (KMO) value was taken as a basis for determining whether the sample size was sufficient for the scale. The determinant value of the correlation matrix was examined to determine whether the data set was factorable or not. The results of Bartlett's Test of Sphericity were used to determine whether the items in the data set were related to each other. Scree-plot methods (scree plot), eigenvalue, and percentage of variance were used to determine the number of factors. CFA was applied to evaluate the suitability of the obtained factors to the data set. In the evaluation of DFA results, fit indices such as root mean square error of approximation (RMSEA), normed fit index (NFI), and relative fit index (RFI) were used. A path diagram was plotted to visualize CFA findings.

## Results

The sociodemographic characteristics of the participants are presented in Table 1. About 583 individuals were included in the study; 286 individuals were male (49.1%) and 297 individuals were female (50.9%). About 264 participants were single (45.3%) and 319 were married (54.7%). The average age of the individuals was 43.16 years, the average height was 168.75 cm, and the average weight was 69.90 kg.

**Table 1:** Sociodemographic characteristics of the participants

Variables	Min-max	Mean±std
Age (age)	18-65	43.16±15.297
Weight (kg)	45-105	69.90w±11.399
Height (cm)	150-191	168.75±9.779
	Variables	n (%)
Gender	Male	286 (49.1%)
	Female	297 (50.9%)
Marital status	Single	264 (45.3%)
	Married	319 (54.7%)

Validity

Content Validity

For each item of the SDF, the CVI of the items was found with the EEF filled out by the experts. About 260 items with CVI less than 0.80 were removed. The total number of items was 65.

Construct Validity

To determine the construct validity of the PPPAS, the Kaiser-Meyer-Olkin (KMO) sample adequacy test was applied to test whether the sample size before EFA was suitable for factorization. Bartlett Test of Sphericity was applied to determine the suitability of the data set for factor extraction. The KMO sample coefficient value was found to be 0.947. Bartlett's value was found as χ^2^=10247.880.

Principal component analysis was used to determine the factor structure of PPPAS. The maximum variability method (varimax) was used as the factor rotation method. The distribution of the items to the factors and their joint load values are shown in Table 2. When the distribution of the items to the factors and the common load values were examined, 17 items were collected in the first factor, 8 items in the second factor, 3 items in the third factor, and 2 items in the fourth factor. As a result of the rotation process applied with the four factors, there were no items with a factor load value of less than 0.30 and no overlapping items with a difference of less than 0.10 between the load values of the two factors.

**Table 2:** Rotated factor matrix

Items	Factor 1	Factor 2	Factor 3	Factor 4
1	0.755	-0.035	0.026	0.089
2	0.744	-0.017	0.009	0.012
3	0.729	0.055	0.081	0.040
4	0.740	0.112	0.045	0.097
5	0.770	0.050	0.007	-0.003
8	0.750	0.066	0.019	0.040
9	0.750	0.000	-0.004	-0.083
11	0.744	0.062	0.032	0.029
12	0.758	0.040	0.071	0.107
16	0.756	0.015	-0.008	-0.089
17	0.752	0.090	0.100	0.030
18	0.780	-0.032	-0.030	0.031
19	0.782	-0.034	0.034	-0.065
24	0.756	0.000	0.010	0.047
28	0.759	0.105	0.069	0.060
29	0.753	0.035	0.034	0.069
30	0.742	0.007	0.006	-0.005
6	0.029	0.851	-0.018	0.032
14	0.081	0.800	-0.008	-0.037
20	0.061	0.809	0.027	0.058
21	0.010	0.769	-0.014	-0.011
23	-0.026	0.788	-0.051	-0.003
25	0.030	0.804	0.035	0.062
26	0.083	0.827	0.060	0.040
27	0.021	0.812	0.053	0.081
10	0.077	0.035	0.859	-0.018
13	0.025	-0.022	0.867	-0.069
15	0.091	0.043	0.846	0.118
7	0.099	0.028	0.139	0.745
22	0.032	0.111	-0.117	0.764

Since the fourth factor consisted of less than three items (items 7 and 22), this factor was deleted. Item 25 was removed with the reason that it semantically distorted the factor structure within the opinion of the expert team. After removing three items, the scale questions were renumbered, and the scale consisted of 27 items. Exploratory factor analysis was performed again with 27 items.

The maximum variability method (varimax) was used as the factor rotation method to determine the factor structure of the PPPAS. The distribution of 27 items to the factors and their joint load values are shown in Table 3.

**Table 3:** Rotated factor matrix

Items	Factor 1	Factor 2	Factor 3
1	0.758	-0.035	0.032
2	0.745	-0.026	0.015
3	0.730	0.051	0.083
4	0.742	0.125	0.052
5	0.769	0.046	0.006
7	0.750	0.071	0.022
8	0.747	-0.011	-0.003
10	0.744	0.058	0.037
11	0.761	0.042	0.078
15	0.752	0.014	-0.010
16	0.752	0.091	0.097
17	0.781	-0.028	-0.025
18	0.780	-0.033	0.034
22	0.757	0.005	0.012
25	0.761	0.106	0.074
26	0.755	0.045	0.036
27	0.741	0.010	0.005
6	0.029	0.851	-0.013
13	0.078	0.803	-0.002
19	0.061	0.815	0.027
20	0.008	0.775	-0.014
21	0.027	0.796	0.049
23	0.083	0.829	0.063
24	0.022	0.818	0.058
9	0.074	0.030	0.863
12	0.020	-0.031	0.865
14	0.092	0.048	0.851

About 17 items were collected in the first factor, 7 items in the second factor, and 3 items in the third factor. As a result of the rotation process applied to the three factors, the factor structures were found to be appropriate. It was observed that the loads on the first factor varied between 0.730 and 0.781, the second factor loads between 0.775 and 0.851, and the third factor loads between 0.851 and 0.865.

The factors in the scale were named according to the item contents. The first factor was named personal well-being, the second factor was named as domestic physical activity, and the third factor was social constraint. First factor: items in the personal well-being group; consisted of items 1, 2, 3, 4, 5, 7, 8, 10, 11, 15, 16, 17, 18, 22, 25, 26, and 27. Second factor: items in the domestic physical activity group; consisted of items 6, 13, 19, 20, 21, 23, and 24. Third factor: items in the social constraint group; consisted of items 9, 12, and 14.

CFA was applied to determine whether the three-dimensional 27-item structure in the scale was confirmed. The fit index values of the scale are given in Table 4.

**Table 4:** Fit index values χ^2^: Chi-square; P: Statistical significance level; Df: Degree of freedom; RMSEA: Root mean square error of approximation; IFI: Incremental fit index; CFI: Comparative fit index; NFI: Normed fit index; NNFI: Non-normed fit index; GFI: Goodness of fit index; AGFI: Adjusted goodness of fit index; RMR: Root mean square residuals; SRMR: Standardized root mean square residual

Index	Research finding
χ^2^/p-value	752.15/<0.001
Df	321
χ^2^: Df	2.343
RMSEA	0.048
IFI	0.955
CFI	0.954
GFI NFI NNFI	0.912 0.923 0.950
AGFI	0.896
RMR	0.060
SRMR	0.047

**Figure 1:**
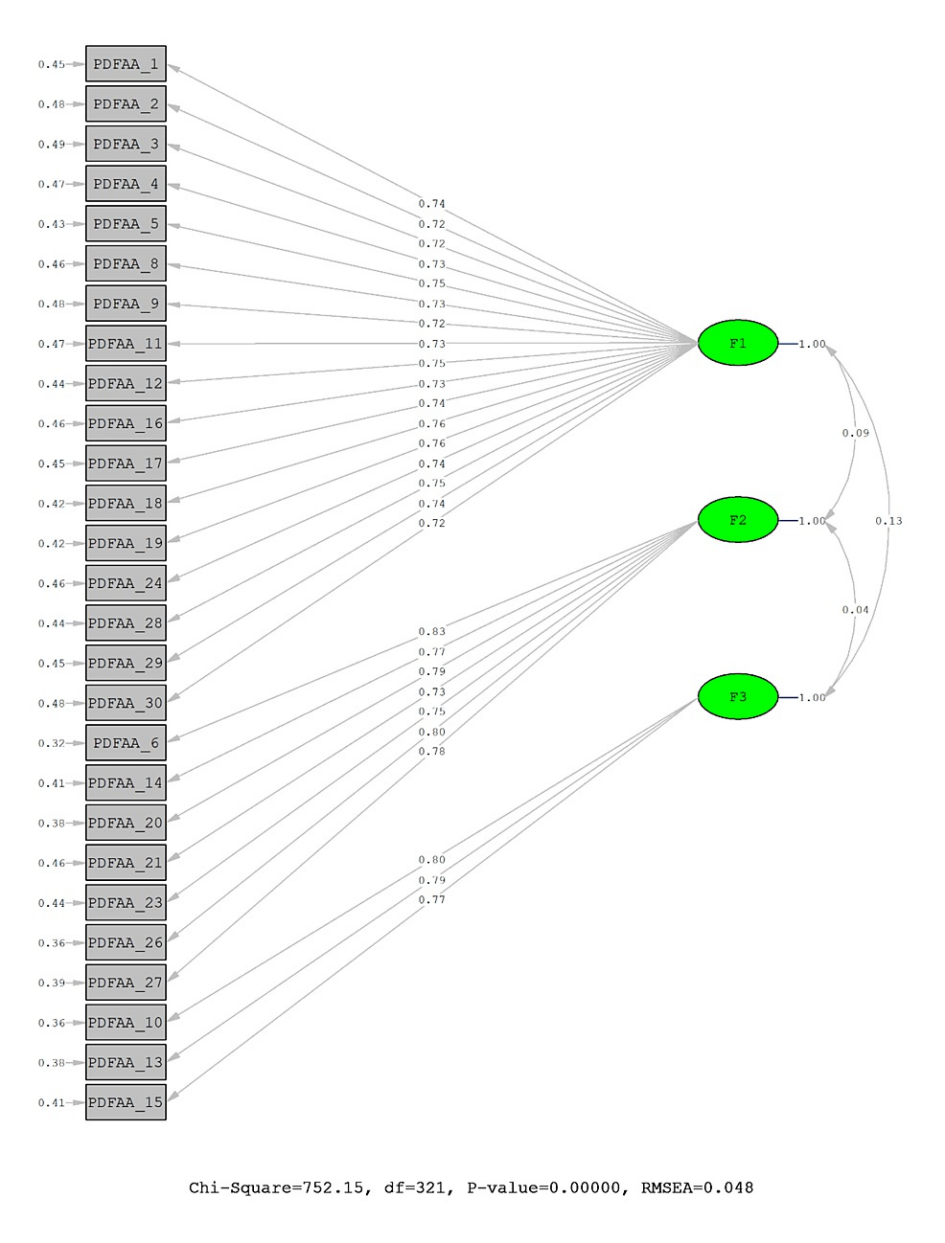
Path diagram for confirmatory factor analysis (F1: personal well-being, F2: domestic physical activity, F3: social constraint) CFA: Confirmatory factor analysis; PPPAS: Pandemic Period Physical Activity Scale; RMSEA: Root mean square error of approximation

Fit indices in the CFA revealed a good fit between data and the scale. All the factor loadings were found above 0.40; ranging from 0.72 to 0.83 and the error values could be named as tolerable since they were calculated to be between 0.32 and 0.49, as a result of CFA suggested (Figure 1).

Reliability

Internal Consistency

The internal consistency reliability of the 27-item scale was evaluated by calculating Cronbach's alpha coefficient. The first factor consisting of 17 items was 0.953; The second factor consisting of seven items was 0.915; The third factor consisting of three items was found to be 0.829. The total Cronbach's alpha coefficient of the scale was found to be 0.912.

Test-Retest

The ICC of PPPAS was calculated as 0.958 (p<0.001). The mean of the first test was 75.38±20.95; the mean of the second test was 77.37±17.08. The test-retest Cronbach's alpha coefficient value was found to be 0.864. When the test-retest analysis results of the sub-factors of the scale were examined, the correlation values of the first factor were 0.963 (p<0.001); the second factor was 0.934 (p<0.001); the third factor was found to be 0.900 (p<0.001).

Parallel Form

IPAQ-SF, EAS, CAS, and TSK were used to determine the parallel form reliability of PPPAS. The total item correlation coefficients between the sub-dimensions of the PPPAS and the sub-dimensions of the EAS are given in Table 5.

**Table 5:** Correlation coefficients of sub-dimensions of Pandemic Period Physical Activity Scale and Epidemic Anxiety Scale *p<0.001 r: Correlation coefficient; p: Statistical significance level; n: Number of participants

		Pandemic Period Physical Activity Scale		
Epidemic Anxiety Scale		Personal well-being	Domestic physical activity	Social constraint
Economic dimension	r	0.104	0.116	0.186
	p	0.023	0.011	<0.001*
	n	479	479	479
Social life dimension	r	-0.583	0.063	0.053
	p	<0.001*	0.171	0.251
	n	479	479	479
Outbreak dimension	r	-0.0047	-0.591	0.013
	p	0.309	<0.001*	0.781
	n	479	479	479
Quarantine dimension	r	-0.053	-0.027	-0.577
	p	0.250	0.562	<0.001*
	n	479	479	479

Correlation coefficients between sub-dimensions were investigated within the context of criterion validity. The correlation between personal well-being with social life dimension was r=-0.583 (p<0.001); the correlation of domestic physical activity with outbreak dimension was r=-0.591 (p<0.001); the correlation between social constraint and quarantine dimension was r=-0.577 (p<0.001).

Criterion Validity

The correlation of PPPAS with similar scales is given in Table 6. The correlation coefficient of PPPAS with similar scales was examined. It was found that PPPAS provided correlation with EAS r=-0.464 (p<0.001); with CAS r=-0.368 (p<0.001); with TSK r=-0.511 (p<0.001).

**Table 6:** Correlation of pandemic period physical activity scale with similar scales *p<0.001 r: Correlation coefficient; p: Statistical significance level; n: Number of participants

		Epidemic Anxiety Scale	Coronavirus Anxiety Scale	Tampa Scale for Kinesiophobia	International Physical Activity Questionnaire Short Form
Pandemic Period Physical Activity Scale	r	-0.464	-0.368	-0.511	0.116
	p	<0.001*	<0.001*	<0.001*	0.005
	n	479	479	479	583

Scoring the Scale

PPPAS has closed-ended (structured) and open-ended (unstructured) questions to determine the level of physical activity. Closed-ended questions are evaluated with a 5-point Likert scale. The answers to the closed-ended questions are arranged as “Strongly disagree,” “disagree,” “undecided,” “agree,” and “strongly agree.” On the scale, “strongly disagree“ is determined as 1 point; ”disagree“ is determined as 2 points; ”undecided“ is determined as 3 points; ”agree“ is determined as 4 points; “strongly agree” is determined as 5 points. Closed-ended questions in the scale consisted of 27 items and items 1, 2, 3, 4, 5, 7, 9, 10, 11, and 14 are reverse coded. In addition, the personal well-being subscale is evaluated over 85 points, the domestic physical activity subscale is evaluated over 35 points, and the social constraint subscale is evaluated over 15 points. The scores that can be obtained from the scale vary between 27 and 135. In the evaluation of the score obtained on the scale, if the average score is close to 27, it indicates that physical activity decreases, and closer to 135 indicates that physical activity increases. Open-ended questions consist of four items and sub-questions of the items.

## Discussion

In this study, we aimed to develop the "Pandemic Period Physical Activity Scale" to measure the physical activity levels of individuals during the pandemic period. Our aim was to develop a scale called the Pandemic Period Physical Activity Scale. As a result of validity and reliability analyses using the following methods, we found that the scale is valid and reliable and can be used to assess physical activity during the pandemic period. The first of these methods was content validity. Content validity is the extent to which the items in the scale serve the purpose of being measured individually or as a whole. The most appropriate way to measure content validity is to submit it to the opinion of experienced experts working in that field. Changes are made to the items in the draft according to the expert's comments [18,19]. An expert team of 11 people consisting of physiotherapists and faculty members who have worked in the field of physical activity was established Davis technique was used for these experts to evaluate and score the items and to calculate the CVI. According to the Davis technique, the number of experts should be at least 3 and a maximum of 20, and items with a CVI less than 0.80 should be excluded from the scale [20]. The expert team evaluated the content validity of 325 items in the SDF with the EEF. As a result of this evaluation, items with a CVI less than 0.80 were excluded from the SDF. In the final stage, the PAF consisting of 58 items was created.

In the scale under development, it helps to have information about the compatibility of the items with the scale by applying a pre-test to a small number of groups representing the sample group [21]. Mertens stated that applying the pre-trial application on a small group of approximately 5% of the target population would be sufficient to reach reliable and valid results [22]. The 58-item PAF was administered face-to-face by the researcher to 221 individuals who met the inclusion criteria as a preliminary trial. With the data obtained as a result of the pre-trial application, the items of the form were evaluated by item analysis; to determine the consistency of the form, internal consistency analysis, which evaluates Cronbach's alpha coefficient, was applied. Nunnally, Cronbach's alpha value should be 0.70 or higher for the scale to be reliable; Liu also said that it should be 0.70 or higher to be considered reliable [23,24]. As a result of the internal consistency analysis, Cronbach's alpha value of PAF was calculated as 0.579 at a low level. Negative values were observed in the total score correlation of some items. About 21 items that reduced reliability were removed from the form and internal consistency analysis was performed again. The Cronbach's alpha value of the 33-item form was found to be 0.770 and a reliable PAF was obtained. The adjusted item-total correlation of PAF ranged from 0.146 to 0.375. The expert team involved in the development of the scale removed three questions because they created confusion. Finally, a highly reliable final PPPAS with 34 questions (30 closed-ended questions, and 4 open-ended questions) was created.

Factor analysis is applied to evaluate the construct validity of the scales and to have information about the sub-dimensions and numbers. While EFA aims to reveal the structure of items in the scale with other items, this model is tested with CFA [25,26]. Factor analysis was performed to determine the construct validity of the data obtained with the PPPAS applied to 583 individuals. Before performing factor analysis, it should be tested whether the data set is suitable for factorization. The suitability of the sample size was determined by KMO; the compatibility of the relationship between the variables is evaluated with the Bartlett test [27]. Sharma said that as a result of the KMO test, values between 0.5 and below are weak; values between 0.5 and 0.6 are moderate; values between 0.7 and 0.8 are good; values between 0.8 and 0.9 are very good; values between 0.9 and 1.0 are excellent [28]. Beavers et al. stated that the KMO test value should be 0.70 and above for EFA to be applied. They said that the Bartlett test should be significant (p<0.001) [29]. In our study, the KMO value was 0.947; the chi-square value (χ^2^)=10247.880 in the result of the Bartlett sphericity test; standard deviation (Df)=435, and the test was found to be significant (p<0.001). The KMO value and the Bartlett sphericity test results showed that the items were quite suitable for factor analysis.

Some criteria were taken into account in calculating the number of factors. The first of these is the table of eigenvalues. Bryman and Cramer said that factors with eigenvalues greater than or equal to one can be indicated as significant in eigenvalue statistics [30]. The other method of calculating the factor number was the scree plot. Thompson stated that the factors at the point where the graph takes a horizontal shape and the slope decreases significantly are considered the maximum number of factors [31]. Costello and Osborne stated that the maximum number of factors was reached when the contribution of each additional factor in explaining the total variance fell below 5% [32]. Maccallum et al. stated that for a factor to be stable, it must have at least three items [33]. As a result of the final EFA performed with 27 items, the first factor (eigenvalue 9.829) explained 36.405% of the variance, the second factor (eigenvalue 4.612) explained 17.083% of the variance, the third factor (eigenvalue was 2.218) explained 8.216% of the variance, while the total variance explained by the three factors together was 61.704%. The maximum variability method (varimax) was used as the factor rotation method. The factor load of the item was found with the varimax technique developed by Kaiser, which is the most preferred factor rotation technique. The size of the factor load of the items means that that item is important for the factor [34]. Comrey and Lee stated that items with a factor load of at least 0.30 should be processed after varimax rotation [35]. As a result of the rotation process applied to the three factors, the factor structures were found to be significant. The loads on the first factor were between 0.730 and 0.781; the second factor loads were between 0.775 and 0.851; The third factor loads were found to vary between 0.851 and 0.865. The factors in the scale were named. In addition, as a result of the EFA applied, PPPAS with 3 factors and 31 items (27 closed-ended questions, 4 open-ended questions) was obtained.

CFA is applied to verify how much the model created as a result of EFA actually measures the structure that it should measure. Along with CFA, there are many fit statistics that evaluate the fit of the models [36]. The fit statistics evaluating the fit of the model as a result of CFA were examined. Chi-square/degree of freedom (χ^2^/df) is the most important measurement value that evaluates the overall fit of the model. Schermelleh-Engel et al. said that a chi-square/degree of freedom below 3 showed a perfect fit for the model, and a model with values between 3 and 8 showed a good fit [37]. In our study, the chi-square/degree of freedom was found to be 2.343, showing that the model fit was excellent. Byrne and Campbell reported that values below 0.05 for RMSEA provide an excellent fit; values below 0.08 provide an acceptable good fit [38]. In our study, the RMSEA value was found to be 0.048, and the model was found to be compatible. Tabachnick and Fidell said that NFI and NNFI values above 0.90 indicated a good fit [39]. In our study, the NFI value was 0.923; The NNFI value was found to be 0.950, and it was seen that the model fit well. Hu and Bentler said that a CFI value of 0.90 and above is a good fit, and a CFI value above 0.95 is a perfect fit [40]. In our study, the CFI value was found to be 0.954, and the model was found to be a good fit. Steiger said that if the GFI and AGFI index values are 0.90 and above, it is an acceptable fit, and if the index values are 0.95 and above, the model provides a perfect fit [41]. In our study, the GFI value was 0.912; The AGFI value of 0.896 was found to be compatible with the model. Simon et al. interpret IFI values of 0.95 and above as perfect fit, and values of 0.85 and above as acceptable [42]. In our study, the IFI value of 0.955 was found to be in perfect agreement with the model. Schermelleh-Engel et al. said that RMR and SRMR values were in excellent agreement if they were 0.05 or less, and within acceptable agreement if they were between 0.05 and 0.1 [37]. In our study, the RMR value was 0.060; The SRMR value of 0.047 was found to be consistent with the model.

The reliability of the scale was evaluated by calculating internal consistency reliability, test-retest reliability, and parallel form reliability. Cronbach's alpha coefficient was taken into account in the internal consistency reliability analysis of the scale. Gliem and Gliem said that the scale is reliable when Cronbach's alpha value is 0.70 and higher [43]. In this study, when the Cronbach alpha coefficients of the factors of the scale were examined, it was found that the first factor consisting of 17 items was 0.953; the second factor consisting of 7 items was 0.915; the third factor consisting of 3 items was found to be 0.829. The total Cronbach's alpha coefficient of the scale was found to be 0.912. The reliability of the scale was found to be high.

The test-retest method was used as another reliable method. Koo and Li stated that for test-retest reliability, the correlation coefficient value should be 0.70 or above, and a close relationship to 1.00 provides a strong correlation [44]. In this study, the intraclass correlation coefficient of the scale was calculated as 0.958 (p<0.001). A high level of significant correlation was observed. When the correlation values of 27 items were examined in the test-retest analysis, it was observed that the values varied between 0.576 and 0.853 (p<0.001). When the test-retest analysis results of the sub-factors were examined, the correlation values of the personal well-being dimension were 0.963 (p<0.001); domestic physical activity dimension 0.934 (p<0.001); the social constraint dimension was found to be 0.900 (p<0.001). The Cronbach's alpha coefficient of the test-retest of the scale was found to be 0.864, quite reliable. These findings revealed the time invariance of PPPAS.

The parallel form method was applied as another reliable method. The comments of Koo and Li were taken into account when examining the correlation values between the scales [44]. Correlation values of sub-dimensions of PPPAS and sub-dimensions of EAS were examined. Correlation coefficients between sub-dimensions were examined. The personal well-being and social life dimension -0.583 (p=0.000); domestic physical activity and outbreak dimension -0.591 (p=0.000); social constraint and quarantine dimension -0.577 (p=0.000) moderate negative correlation found. The correlation coefficient of PPPAS with similar scales was examined. Moderate negative correlation with EAS -0.464 (p<0.001); weak negative correlation with CAS -0.368 (p<0.001); Negative moderate correlation with TSK was -0.511 (p<0.001). It was observed that the increase in anxiety and coronavirus anxiety during the epidemic period decreased physical activity, and the increase in fear of movement decreased physical activity.

The findings of this study are based on data obtained from participants living in only one city. This can be characterized as a limitation of the study. For this reason, studies with larger and different sample groups may be useful in evaluating the validity and reliability of the scale.

## Conclusions

As a result, it was concluded that the Pandemic Period Physical Activity Scale, which consists of 31 items (27 closed-ended questions and 4 open-ended questions), is a valid and reliable measurement tool and can be used to measure the level of physical activity during the pandemic period.
